# A study on different types of moral courage and coping styles of clinical nurses: based on potential profile analysis

**DOI:** 10.1186/s12912-023-01590-5

**Published:** 2023-11-08

**Authors:** Nian Hong, Niu Qichao, Chen Dong, Tai Chunling, Pang Dong, Lv Xinyu, Su Yu, Liu Shilong, Zhang Yuhuan

**Affiliations:** 1 Cancer Radiotherapy Department, Second Affiliated Hospital of Harbin Medical University, Harbin City, 150000 Heilongjiang Province China; 2 Second Affiliated Hospital of Harbin Medical University, Harbin City, 150000 Heilongjiang Province China; 3 Heilongjiang Higher Nursing School, Harbin City, 150000 Heilongjiang Province China; 4 Department of Neurology, Second Affiliated Hospital of Harbin Medical University, Harbin City, 150000 Heilongjiang Province China

**Keywords:** Nurse, Moral courage, Response methods, Potential profile analysis

## Abstract

**Background:**

In professional ethics-related events, there are various unpleasant and complex ethical issues that require strong moral courage. Our aim is to identify and describe the potential categories of moral courage among nurses and to clarify the coping styles of nurses under different categories.

**Method:**

A cross-sectional study was conducted using three data collection tools: a self-designed general information questionnaire, a Chinese version of the Moral Courage Scale, and a Trait Coping Style Questionnaire. Three hundred fourteen nurses from a tertiary hospital in Heilongjiang Province, China, were analysed using potential profile analysis, descriptive analysis, and Mann-Whitney U test data.

**Result:**

The latent profile analysis (LPA) results indicate that the two-profile model is the most suitable and supports the existence of two different moral courage profiles: the low moral courage group (60.51%) and the high moral courage group (39.49%), with a high relative entropy value (0.922). The results point to a good profile solution, and there are significant differences between the two profiles. The Mann-Whitney U-test results showed that the positive coping scores of the high moral courage group were significantly higher than those of the low moral courage group, and the negative coping scores of the high moral courage group were significantly lower than those of the low moral courage group.

**Conclusion:**

Our results reveal the heterogeneity of moral courage in the nurse sample and indicate that nurses in the high moral courage group tend to choose positive coping styles, while nurses in the low moral courage group are more likely to develop negative coping emotions. This provides important significance and reference value for nursing managers, who can propose customised management plans based on the types of moral courage of the nursing community and the coping styles under different categories.

## Background

The ‘Outline of the Development Plan for China’s Nursing Industry from 2016 to 2020’ emphasises the importance of adopting a patient-centred service concept and promoting high-quality nursing services [1]. This entails that nurses must not only focus on patients’ physical health but also prioritise their psychological safety. As an illustration, a nurse’s professional obligation includes all “technical procedures” that they perform, such as resuscitation or wound care, for she or he has the proper education and competence to carry out them [2, 3]. But nurses have a duty in the nurse-patient interaction. This responsibility makes nursing an ethical enterprise [4]. It covers not just psychological care but much more. For instance, nurses must give patients the most fundamental humanistic care, pay attention to their own emotions, act in the patient’s best interests, feel the patient’s needs and inner suffering, speak up when the patient’s rights are violated, protect the patient’s dignity, etc. [5, 6]. Under such care requirements, it means that nursing work is a moral practice [7].

Moral courage is the ability to do the right thing even when there are risks or when someone else has more power than you [8]. Moral courage is an important virtue in nursing work because it helps nurses expand their social and moral space, solve moral problems, keep their moral purity, and grow as professionals. Making sure they act in a responsible way and giving good care are all important parts [9–12]. Nurses need to develop courage and sensitivity to meet the challenges of their profession, which include protecting patient privacy, caring for infected patients, helping dying or poor patients, giving invasive care, dealing with newly found diseases and emergencies, and advocating for patients [8, 13, 14].

As is well known, nursing is a highly stressful profession [15]. Fear of unfriendly reactions from coworkers, unemployment, violence, and lower pay may all cause nurses to stop acting ethically in this complex clinical setting. This can lead to moral distress, depression, guilt, anger, feeling helpless, and feeling like they aren’t worth anything [16]. Many studies have shown that nurses often face moral problems [17–20]. This is an issue that can’t be ignored. Existing research surveys have shown that some nurses have high scores for moral courage while others have low scores, indicating that moral courage is not entirely homogeneous among nurses [21, 22]. However, few studies have explored the categories of moral courage in the nursing community, which is one of the purposes of this study.

Coping has been shown to be a stable psychological and behavioural approach that helps people deal with both outside and inside problems [23]. Coping is defined as a set of cognitive and behavioural techniques employed by individuals in stressful situations to handle internal and external requirements, with good and negative coping styles being diametrically opposed [24]. Individuals who focus more on problems and respond to stress through tactics such as problem-solving, seeking social support, and cognitive reconstruction are examples of positive coping [25]. Negative coping, on the other hand, is characterised by the use of more emotional and palliative coping technique [26].

Based on the perspective of psychological resilience, individuals with more positive traits are more able to mobilize internal and external resources to solve problems in the face of stressful events [19]. Based on this theory, it is speculated that nurses who actively respond well can make full use of their resources in the face of stressful events related to professional ethics, mobilize the resources at their disposal to solve problems, and may improve their moral courage when dealing with difficult ethical issues. Nurses who actively respond poorly tend to have a negative understanding of professional ethics-related events and are not good at integrating and utilizing their own resources, which can lead to a negative work attitude, deepening negative cognition and evaluation of their work, and a natural decline in moral courage and even moral indifference. In short, those with high moral courage belong to positive actors, while those with low moral courage belong to negative actors. However, through a literature review, few studies have pointed out the coping styles of different categories of nurses’ moral courage.

On the basis of clarifying the potential differences in nurses’ moral courage, this study investigates the coping styles of individuals with different types of moral courage in order to provide a theoretical foundation and practical recommendations for enhancing nurses’ moral courage in accordance with the characteristics of different clinical nurse groups.

## Object and method

This is a cross-sectional investigation. Using a simple sample method [27], this study chose in-service nurses from a tertiary A hospital in Harbin, China, as research subjects. Inclusion Criteria: 1) Possession of a valid nursing licence; 2) Informed consent and willingness to engage in this study voluntarily. Criteria for exclusion: 1) nurses enrolled in continuing education and nursing students; 2) nurses unable to participate in the survey due to personal absence, illness, maternity leave, or because they are pursuing additional education or studying.

Sample size calculation: According to the sample size estimation formula *N* = [Max (number of items)] (5–10), plus a 10% invalid sample size, The main scale of this study, the Moral Courage Scale, consists of 21 items with a sample size of 116–231.

### Survey Tools

#### General information questionnaire

Gender, age, department, length of service, professional title, education, marital status, monthly salary, night shift frequency, and personnel interactions are among the ten indicators developed by the researchers.

#### Chinese version of the Nurses’ moral courage scale

Using Wang Siyao’s [28] Chinese version of the Nurses’ Moral Courage Scale (NMCS) to evaluate clinical nurses’ self moral courage. Permission to use the NMCS was obtained from the copyright holder [29]. The scale consists of 4 dimensions, 21 items, including ethics (7 items), commitment to good care for patients (5 items), compassion and real presence with patients (5 items), moral responsibility (4 items), and the Liket5 scoring method. “Completely inconsistent with me” = 1, “slightly inconsistent with me” = 2, “somewhat consistent with me” = 3, “relatively consistent with me” = 4, “exactly match me “=5. The total score of the scale is 21–105 points, and the higher the score, the higher the moral courage of the nurse. The Cronbach’s a coefficient of the Chinese version of NMCS was 0.967, and the Cronbach’s a coefficients of each dimension were 0.885, 0.894, 0.905, and 0.890, all > 0.70. The correlation coefficient between the total score and each item was 0.585 to 0.875 (*P* < 0.01), the KMO value was 0.953, and the approximate value of the Barrett spherical test was x^2^ = 6396.482 (*P* < 0.001), indicating good reliability and validity.

#### Trait Coping Style Questionnaire

The questionnaire consists of two dimensions: positive coping style and negative coping style. Each dimension has 10 items and adopts a 5-level scoring method. Scores of 5, 4, 3, 2, and 1 indicate strong agreement, agreement, neutrality, disagreement, and strong disagreement, respectively [30]. Used to reflect the coping traits of participants when facing difficulties and setbacks, the higher the score, the more obvious the coping characteristics of the population. The Cronbach’s a coefficient of the Chinese version of TCSQ was 0.911, and the Cronbach’s a coefficients of each dimension were 0.833 and 0.829, all > 0.70. The correlation coefficient between the total score and each item was 0.512 to 0.689 (*P* < 0.01), the KMO value was 0.895, and the approximate value x^2^ = 4242.511 (*P* < 0.001) in the Barrett spherical test showed good reliability and validity.

### Survey methods

Researchers modified the survey questionnaire for use on the Questionnaire Star platform (https://www.wjx.cn/vj/PRuTQS0.aspx), introducing the objective, significance, and expected time to finish the survey in the beginning interface. The questionnaire measurement takes about 10 min to complete and includes three research tools. After obtaining the consent of the head of the nursing department, the questionnaire link is forwarded through the head nurse of each department. Nurses voluntarily participate, click on the link to fill in, and all options are set to anonymous. The questionnaire option is required and once completed, it will be directly submitted to the website backend, and the head nurse does not have the authority to view it. Throughout the entire question answering process, the head nurse is only responsible for forwarding the online questionnaire answer link and does not participate in the questionnaire collection process.

### Statistical methods

The obtained data was analysed using SPSS 21.0 and Mplus 8.3 software. Firstly, LPA was used to determine the potential categories of moral courage among clinical nurses. The commonly used adaptation and fitting indicators include the Akaike Information Criterion (AIC), the Bayesian Information Criterion (BIC), and the sample corrected BIC (aBIC). The smaller the values of these three indicators, the better the fit of the data to the model. The range of Entropy values is 0–1, and a larger entropy value indicates a higher accuracy of classification. When the *P*-values corresponding to the Bootstrap Likelihood Ratio Test (BLRT) and Lo Mendell Rubin Likelihood Ratio Test (LMR) based on the Bootstrap method are significant (*P* < 0.05), it indicates that the k-class model performs better than the k-1 class model. Secondly, SPSS 23.0 was used for the statistical analysis of the data. Check the normality of the data distribution using the Kolmogorov-Smirnov (KS) test (*P* < 0.05). The counting data are expressed in frequency and percentage, and the median and quartile interval of the measurement data are expressed. The Mann-Whitney U test was used for intergroup comparative analysis. The difference was statistically significant at *P* < 0.05.

## Results

### General information of nurses

Refer to Table 1 for specifics.

**Table 1:** General information of nurses (*n* = 314)

project	classification	Number of cases (Composition ratio)
Gender		
	male	19 (6.1%)
	female	295 (93.9%)
Age		
	22–30	107 (34.1%)
	31–40	179 (57.0%)
	41–53	28 (8.9%)
Department		
	internal medicine	124 (39.5%)
	Surgery	107 (34.1%)
	ICU and operating room	69 (22.0%)
	Other ①	14 (4.5%)
Years of Work		
	1–10 years	185 (58.9%)
	11–20 years	104 (33.1%)
	21–37 years	25 (8.0%)
title		
	Nurse	32 (10.2%)
	Senior nurse	186 (59.2%)
	Supervisor nurse or above ②	96 (30.6%)
education		
	specialty	22 (7.0%)
	undergraduate	276 (87.9%)
	graduate student	16 (5.1%)
marital status		
	unmarried	75 (23.9%)
	married	234 (74.5%)
	divorce	5 (1.6%)
Average monthly income (yuan)		
	<5000	119 (37.9%)
	5001–10,000	174 (55.4%)
	> 10,001	21 (6.7%)
Night shift frequency		
(Monthly)	0 times	72 (22.9%)
	1–5 times	138 (43.9%)
	6–10 times	91 (29.0%)
	11–15 times	13 (4.1%)
Personnel Relations		
	Contract system or personnel agency	253 (80.6%)
	Officially under preparation	61 (19.4%)

① Other departments include Pediatric Radiology Interventional Department and Rehabilitation Department; ② The supervisor nurse or above includes 8 deputy chief nurses and 2 chief nurses

### Description of moral courage, positive coping, and negative coping scores

The total scores for moral courage and moral integrity, commitment to good care for patients, compassion and being truly with patients, and moral responsibility were 42.00 (31.00, 57.00), 14.00 (10.00, 19.00), 11.00 (8.00, 15.00), 10.00 (6.00, 13.00), and 8.00 (5.00, 11.00), respectively. Refer to Table 2 for specifics.

**Table 2:** Score of Moral Courage, Positive Coping, and Negative Coping (*n* = 314)

variable	minimum value	Maximum value	[M(P25,P75)]
Total score of moral courage	21	104	42.00 (31.00, 57.00)
Ethical Conduct	7	34	14.00 (10.00, 19.00)
Commitment to Good Care for Patients	5	25	11.00 (8.00, 15.00)
Compassion and true presence with patients	5	25	10.00 (6.00, 13.00)
Moral responsibility	4	20	8.00 (5.00, 11.00)
Actively respond	10	50	33.00 (30.00, 38.00)
Negative coping	10	50	32.00 (30.00, 36.00)

### Potential profile analysis of moral courage in clinical nurses

On the basis of the four dimensions of moral courage: moral integrity, commitment to excellent care for patients, compassion and real presence with patients, and moral responsibility, one to three potential category models were developed. Table 3 displays the results of LPA model fitting indicators. From model 1 to model 3, AIC, BIC, and aBIC decreased gradually, while entropy increased gradually; however, the LMR of model 3 is greater than 0.001, indicating that there is no classification significance. Consequently, the moral courage of clinical nurses was ultimately classified into two distinct categories.

**Table 3:** Potential Profile Fitting Index for Moral Courage of Clinical Nurses (*n* = 314)

Model	LL	AIC	BIC	aBIC	LMR	BLRT	Entropy	Category probability
1	− 3588.136	7192.273	7222.268	7196.894	–	–	–	1
2	− 3196.983	6419.965	6468.707	6427.475	0.0000	0.0000	0.922	0.60510/ 0.39490
3	− 2997.721	6031.442	6098.931	6041.84	0.0578	0.0000	0.936	0.41401/ 0.27389/0.31210

Table 4 displays the probabilities of belonging to the two prospective categories. The probabilities of nurses’ moral virtue (rows) belonging to each potential category (columns) range from 96.3 to 98.9% on average, indicating that the results of classifying into two potential categories are reliable.

**Table 4:** Average Attribution Probability of Participants (Rows) in Each Potential Profile (Column)

	C1	C2
C1	0.989	0.011
C2	0.037	0.963

### Characteristics, distribution, and naming of potential categories of moral courage among nurses

**Fig. 1:**
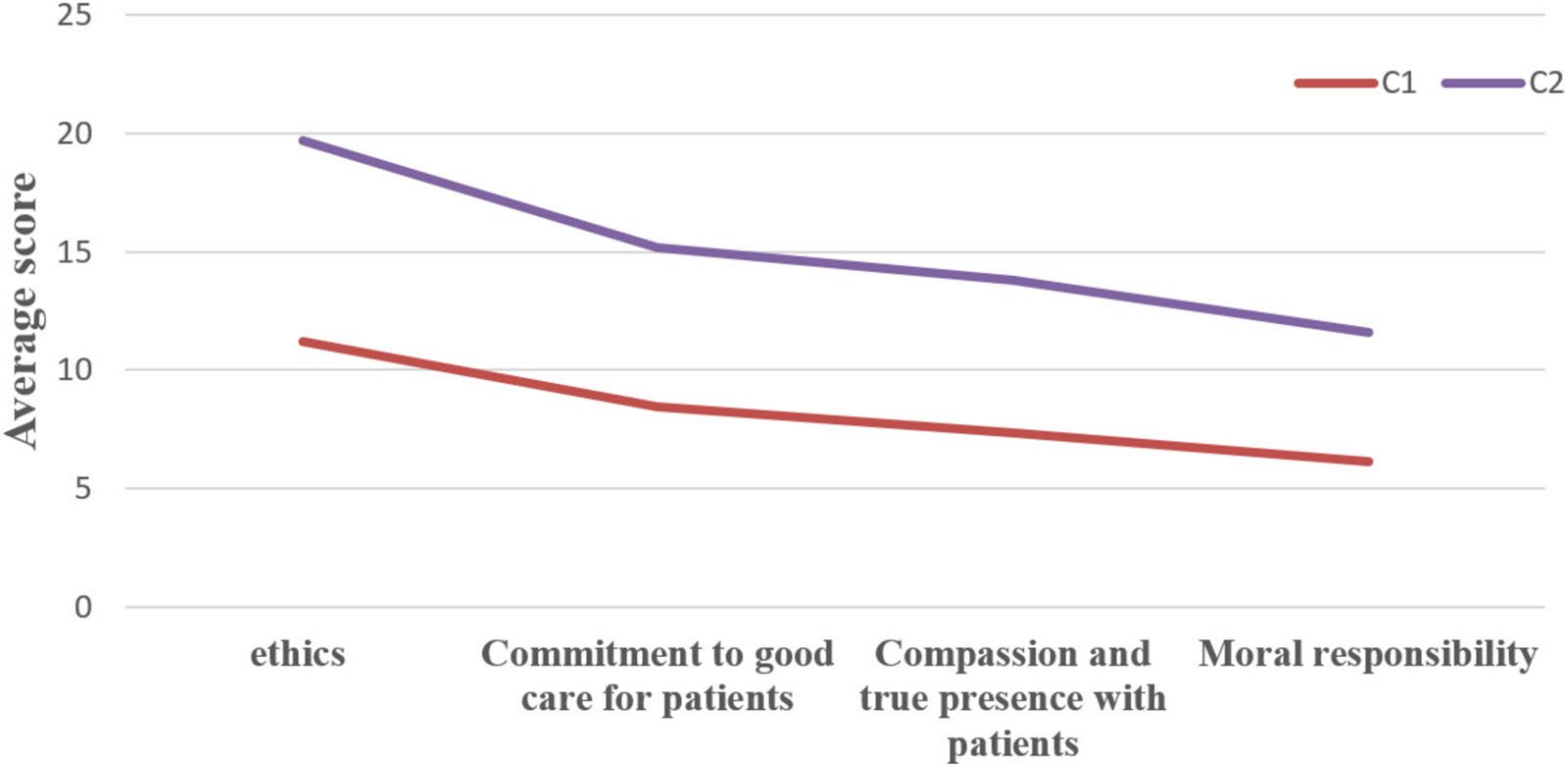
Distribution of Characteristics of Two Potential Categories of Moral Courage in Clinical Nurses

The average values of the two categories on the four dimensions of the Clinical Nurse Moral Courage Questionnaire are depicted in Fig. 1 based on the classification results of potential categories. The mean difference between the two potential categories of nurses in four dimensions is statistically significant, and the overall morphological trend of the two subtypes is consistent. In the latent category group, the scores of all dimensions in the C1 category are lower than those in the other categories, with 190 individuals representing 60.51% of the total population. Consequently, it is referred to as the “Low Moral Courage Group.” The C2 category scored higher than C1 in all dimensions, with 124 individuals comprising 39.49% of the total. Therefore, it was dubbed the “High Moral Courage Group.”

### Comparison of moral courage among different nurses

Intergroup analysis was conducted using the form of moral courage as the independent variable and the four dimensions and total score of moral courage as the dependent variables. As shown in Table 5, there were statistically significant differences between the two categories of moral courage across all dimensions.

**Table 5:** Differences in moral courage and various dimensions among different types of nurses (*n* = 314)

Items	C1	C2	Z	*P*
Ethical Conduct	11.00 (7.75,14.00)	20.00 (17.00,21.00)	−13.941	0.000
Commitment to Good Care for Patients	9.00 (5.00,10.00)	15.00 (14.00,16.00)	−14.507	0.000
Compassion and true presence with patients	7.00 (5.00,10.00)	14.00 (12.00,15.00)	−13.957	0.000
Moral responsibility	6.00 (4.00,8.00)	12.00 (10.00,12.00)	−14.285	0.000
Total score of moral courage	35.00 (23.00,42.00)	59.50 (54.00,63.00)	−14.983	0.000

### Comparison of coping styles among nurses with different types of moral courage

Intergroup comparisons were conducted using the type of moral courage of nurses as the independent variable and positive and negative coping as the dependent variables. Table 6 shows that there were significant differences between the two categories of moral courage that nurses displayed in terms of coping style scores. Positive coping scores of the group with high moral courage were significantly higher than those of the group with low moral courage; negative coping scores of the group with high moral courage were significantly lower than those of the group with low moral courage.

**Table 6:** Comparison of Nurse Coping Style Scores for Each Potential Profile

Items	CI	C2	Z	*P*
Actively respond	34.00 (31.00,39.00)	32.50 (32.00,35.00)	−3.727	0.000
Negative coping	32.00 (30.00,37.00)	60.00 (62.00,69.00)	−2.428	0.015

## Discussion

This study used latent profile analysis to identify two different potential categories in the four dimensions of nurses’ moral courage, namely the high moral courage group and the low moral courage group. There were significant differences in the total moral courage score and different dimension scores among each group, indicating the heterogeneity of nurses’ moral courage. In addition, this study found significant differences in positive and negative coping scores among potential categories of moral courage, which to some extent confirms the effectiveness of the two potential categories. From the distribution of various types, nurses in the low moral courage group accounted for 60.51% of all participants, while nurses in the high moral courage group accounted for 39.49%. The overall moral courage score of nurses is 42.00 (31.00, 57.00) points, with a maximum total score of 104 points. Overall, it indicates that the moral courage of clinical nurses is at a lower average level, lower than the research results of domestic and foreign scholars [14, 22, 31, 32].

There are two possible explanations for the low moral courage score of nurses in this study: first, intrinsic factors within the nurses themselves may be to blame. In clinical work, nurses not only take care of diseases but also undertake various tasks such as daily life care, psychological counseling, and health education to take care of patients. This requires nurses to always question their moral integrity and the original intention of choosing a profession and maintain moral sensitivity, but the combination of factors such as a continuous high-pressure work environment and fatigue may lead to nurses refusing to engage in ethical behavior [33]. Second, the external environmental factors to which nurses are exposed, such as the professional level system, the reward and punishment incentive system, and the empowerment and advice system within healthcare, increase the uncertainty with which nurses deal with professional ethics issues and diminish their moral courage when confronting ethical issues [34].

In addition, the negative coping scores of the high moral courage group and the low moral courage group gradually increase, whereas the positive coping scores of the low moral courage group are the lowest, indicating that the low moral courage group is more likely than the high moral courage group to develop negative coping emotions. Studies have shown that individuals with negative coping styles frequently exhibit distorted thinking, negative evaluations, and incorrect self-evaluations (such as feeling powerless to solve problems). They attempt to avoid stressful situations by concentrating on negative means of minimising discomfort as much as possible [26, 35]. Therefore, nurses with a negative coping style will experience a sense of discord when confronted with moral issues, which will diminish their moral courage [36]. Positive coping, on the other hand, can enhance an individual’s effective response to challenges and imbue stressful events with positive significance [37–39]. Therefore, nurses who confront moral dilemmas with extreme adaptability have a constructive problem-solving coping style, which encourages them to exhibit a high level of moral courage.

### Research significance and clinical guidance

This study used latent profile analysis to identify two different potential categories in the four dimensions of nurses’ moral courage, namely the “high moral courage group” and the “low moral courage group”. According to the findings of this study, the moral courage of nurses is below average. Importantly, this is the first study to investigate the group heterogeneity of nurses’ moral courage and classify it into two potential categories using scientific methods, thereby overcoming some of the limitations of the variable-centred research approach. Second, for the first time, discuss the coping styles of nurses in various moral courage categories.

For clinical practise guidance, new guidance and ideas for nursing managers can be emphasised from three perspectives: first, by reminding nursing managers to value the moral courage of nurses. Specifically, by establishing an example of moral courage, sharing experiences of multi-professional cooperation in resolving moral and ethical issues, and encouraging nurses to accept their own flaws and identify ethical issues with sensitivity, this objective will be achieved. Make decisive decisions and accept responsibility; actively seek assistance when ethical conflicts arise; overcome a dread of conflict; and become an advocate for patient rights [22, 40]. On the other hand, it is recommended that nursing managers emphasise the significance of affirmative responses.

Specifically, employees can receive regular training on pertinent information to enhance their ability to self-regulate, maintain psychological equilibrium, and manage various ethical dilemmas at work effectively [41, 42]. Moreover, nursing administrators should differentiate the prospective distribution of moral courage among nurses and consider the coping styles of nurses within each category. Taking a dual approach, moral courage and coping styles should be considered concurrently, and more targeted and appropriate management techniques and reward and punishment systems should be specified. Perhaps this will better motivate nurses’ internal positive forces, encourage the development of nurses’ moral courage, and encourage nurses to be courageous patient guardians.

### Limitations

This study, like all other types of research, has its limitations. This study focused solely on the coping characteristics of moral courage groups; in the future, additional research variables can be added to obtain a more complete understanding of the characteristics of moral courage groups. In addition, survey tools are self-evaluation tools that may lead to information bias and, to some extent, limit the representativeness of the results.

## Summary

The study used latent profile analysis to find two potential categories of nurses’ moral courage, namely the “low moral courage group” and the “high moral courage group”, and clarified the coping characteristics of nurses in different moral courage categories, providing new intervention perspectives for nursing managers, namely fully considering the potential categories of nurses’ moral courage and developing targeted intervention strategies based on the coping characteristics.

## Data Availability

The dataset of this study can be obtained from suitable authors under reasonable conditions. Data contact Zhang Yuhuan, email 2802262584@qq.com.
